# Proteomics Approach Highlights Early Changes in Human Fibroblasts-Pancreatic Ductal Adenocarcinoma Cells Crosstalk

**DOI:** 10.3390/cells11071160

**Published:** 2022-03-29

**Authors:** Verena Damiani, Maria Concetta Cufaro, Maurine Fucito, Beatrice Dufrusine, Claudia Rossi, Piero Del Boccio, Luca Federici, Maria Caterina Turco, Michele Sallese, Damiana Pieragostino, Vincenzo De Laurenzi

**Affiliations:** 1 Department of Innovative Technologies in Medicine and Dentistry, University “G. d’Annunzio” of Chieti-Pescara, 66100 Chieti, Italy; verena.damiani@unich.it (V.D.); maurine.fucito@unich.it (M.F.); beatrice.dufrusine@unich.it (B.D.); claudia.rossi@unich.it (C.R.); luca.federici@unich.it (L.F.); michele.sallese@unich.it (M.S.); delaurenzi@unich.it (V.D.L.); 2 Center for Advanced Studies and Technology (CAST), University “G. d’Annunzio” of Chieti-Pescara, 66100 Chieti, Italy; maria.cufaro@unich.it (M.C.C.); piero.delboccio@unich.it (P.D.B.); 3 Department of Pharmacy, University “G. d’Annunzio” of Chieti-Pescara, 66100 Chieti, Italy; 4 Department of Psychological, Health and Territory Sciences, School of Medicine and Health Sciences, University “G. d’Annunzio” of Chieti-Pescara, 66100 Chieti, Italy; 5 Department of Medicine, Surgery and Dentistry Schola Medica Salernitana, University of Salerno, 84081 Baronissi, Italy; mcturco@unisa.it; 6 R&D Division, BIOUNIVERSA s.r.l., 84081 Baronissi, Italy

**Keywords:** fibroblasts activation, PDAC microenvironment, proteomics, ingenuity pathway analysis

## Abstract

Pancreatic ductal adenocarcinoma (PDAC) is a leading cause of cancer mortality worldwide. Non-specific symptoms, lack of biomarkers in the early stages, and drug resistance due to the presence of a dense fibrous stroma all contribute to the poor outcome of this disease. The extracellular matrix secreted by activated fibroblasts contributes to the desmoplastic tumor microenvironment formation. Given the importance of fibroblast activation in PDAC pathology, it is critical to recognize the mechanisms involved in the transformation of normal fibroblasts in the early stages of tumorigenesis. To this aim, we first identified the proteins released from the pancreatic cancer cell line MIA-PaCa2 by proteomic analysis of their conditioned medium (CM). Second, normal fibroblasts were treated with MIA-PaCa2 CM for 24 h and 48 h and their proteostatic changes were detected by proteomics. Pathway analysis indicated that treated fibroblasts undergo changes compatible with the activation of migration, vasculogenesis, cellular homeostasis and metabolism of amino acids and reduced apoptosis. These biological activities are possibly regulated by ITGB3 and TGFB1/2 followed by SMAD3, STAT3 and BAG3 activation. In conclusion, this study sheds light on the crosstalk between PDAC cells and associated fibroblasts. Data are available via ProteomeXchange with identifier PXD030974.

## 1. Introduction

Pancreatic ductal adenocarcinoma (PDAC) accounts for over 90% of all pancreatic cancers and remains one of the leading causes of cancer mortality worldwide [1,2]. The outcome of PDAC treatments is highly dependent on the stage of the disease, although overall the 5-year survival rate is only 8%. Unfortunately, approximately 80–90% of PDAC cases are detected at an advanced stage and have distal metastases, mainly because this disease triggers nonspecific symptoms and no biomarkers are available at an early stage. Systematic chemotherapy is the first line of intervention for these patients, whereas surgery and chemotherapy are the standard care for patients with early stages resectable tumors [3,4].

Additional reasons for the poor prognosis of PDAC include disease heterogeneity, immunosuppressive and desmoplastic tumoral microenvironment [5,6]. PDAC stratification is complex and the best way to perform it is unclear, which impacts the identification of the best treatment options. Macrophages and granulocytes recruited at the tumor site mediate an immunosuppressive activity towards T cells and thus impair an appropriate antitumor immune response. Furthermore, immunosuppressive T cell subpopulations (e.g., γδ T cells) have also been documented in PDAC tumors [7]. PDAC tumor masses, besides immune and cancer cells, contain large amounts of desmoplastic stroma secreted by activated fibroblasts known as cancer associated fibroblasts (CAFs) [8,9]. Normal quiescent fibroblasts, upon different stimuli, can be recruited and activated inside the tumor. The transition from normal fibroblasts to CAFs begins in the early stages of tumorigenesis and progresses with the tumor [10,11,12]. CAFs are the most abundant cells in PDAC and multiple studies have investigated how the tumor-stroma crosstalk leads to tumor progression, invasion, metastasis and chemoresistance [13]. Activated fibroblasts exhibit excessive secretory and remodeling extracellular matrix (ECM) phenotypes, are more migratory and vulnerable to epigenetic modifications, enabling their function as precursors for different cell types. Nevertheless, CAFs are an ill-defined heterogeneous cell population whose activation can be recognized by the expression of vimentin, platelet-derived growth factor (PDGF) and/or alpha-smooth muscle actin (alpha-SMA) [14].

The secretion of collagen, laminin and fibronectin creates a firm extracellular matrix that contributes to the poor vascularization of the PDAC, thus compromising the delivery of drugs into the tumor mass and the effectiveness of the treatment [15,16]. Insufficient vascularization also leads to hypoxia, an important component of the PDAC microenvironment, which further stimulates CAFs to release matrix proteins and a large repertoire of soluble factors capable of affecting cancer cells. In fact, particular subtypes of CAFs can influence the aggressiveness of cancer cells through paracrine crosstalk with any PDAC cell [13]. The interaction between tumor cells, immune cells and CAFs involves a wide range of molecules including cytokines such as transforming growth factor beta (TGFB), interleukin-6 (IL6), C-C motif chemokine ligand 2 (CCL2), interleukin-8 (IL8), C-X-C motif chemokine ligand 1 (CXCL1) and hepatocyte growth factor (HGF) [17].

Given the importance of fibroblast activation in PDAC pathology, it is critical to define the mechanisms involved in the early transformation of normal fibroblasts. These cells within tumors are persistently activated by tumor cells. In response, fibroblasts secrete soluble factors that modulate the surrounding tumor microenvironment and regulate stroma-cancer interactions [18]. In the present study, firstly, we identified the proteins released from the pancreatic cancer cell line MIA-PaCa2 by proteomic analysis of conditioned medium (CM). Subsequently, normal fibroblasts were treated for 24 h and 48 h with this CM and both the secretome and the proteostatic changes within the fibroblasts were investigated by proteomics. Pathway analysis of the proteomics indicated that integrin beta 3 (ITGB3) and TGFB1/2 could be key players in increasing fibroblast movement and inhibiting apoptosis followed by the activation of mothers against decapentaplegic homolog 3 (SMAD3), signal transducer and activator of transcription 3 (STAT3) and Bcl2-associated athanogene 3 (BAG3), important molecules involved in chemotaxis, cell adhesion and actin cytoskeleton signaling.

## 2. Materials and Methods

### 2.1. Cell Lines

The human pancreatic ductal adenocarcinoma cell line MIA-PaCa2 was purchased from ATCC (Manassas, VA, USA) and cultivated in Dulbecco’s modified Eagle’s medium (DMEM, Gibco, Thermo Fisher Scientific, Waltham, MA, USA), supplemented with 10% fetal bovine serum (FBS, Gibco, Thermo Fisher Scientific). Human dermal fibroblasts (HDFs, MERCK KGaA, Darmstadt, Germany) were cultured in fibroblast growth medium (MERCK KGaA) without FBS supplement. Both cell lines were grown at 37 °C and 5% CO_2_. HDFs were used between passages 3–10. CAFs were purchased from Neuromics (Edina, MN, USA) and cultured in MSC-GRO (VitroPlus III, low serum, complete) from Neuromics.

### 2.2. Production of MIA-PaCa2 Cells Conditioned Medium (CM)

After thawing, MIA-PaCa2 was grown for three passages before seeding for supernatant collection. Cells were seeded on a 10 cm^2^ tissue culture dish at a density of 3 × 10^6^ in supplemented DMEM. After 24 h, the medium was removed, and adherent cells were washed twice with phosphate-buffered saline (PBS). After that, 10 mL of fresh DMEM was added to the cells. MIA-PaCa2 CM was collected 24 h later and centrifuged at 4000 rpm for 10 min at room temperature (RT) to pellet cell debris. The supernatant was directly used for HDF treatment.

### 2.3. Collection of HDFs after MIAPaCa2-CM Treatment for Proteomic Analysis

A total of 400,000 HDFs were seeded on 6 cm^2^ tissue culture dishes in fibroblast growth medium. After 24 h, cells were washed twice with PBS, and fresh DMEM serum-free (SF) or MIA PaCa-2 CM was added for a further 24 or 48 h. After treatment, supernatants were removed and centrifuged at 4000 rpm for 10 min at RT and stored at −80 °C for further analysis. Instead, HDFs were washed once with PBS and collected using a cell scraper and the cell suspensions were transferred into 1.5 mL tubes. Then, cells were centrifuged at 2000 rpm for 5 min at RT. After media removal, the cell pellet was immediately stored at −80 °C.

### 2.4. Label-Free Proteomics Analysis

In order to evaluate the protein expression of HDF cells at different times of exposure to MIA-PaCa2 CM, we performed a comparative shotgun proteomics analysis of both HDF cells and their respective supernatants after 24 and 48 h. At the end of treatments, samples were prepared for the Filter Aided Sample Preparation (FASP) protocol. Regarding cellular pellets, they were lysed by sonication on ice (Sonicator U200S control, IKA Labortechnik, Staufen, Germany) at 70% amplitude in a lysis buffer (urea 6 M in 100 mM Tris/HCl, pH = 7.5). HDF lysed cells were quantified through a Bradford assay (Bio-Rad, Hercules, CA, USA) using Bovine Serum Albumin (BSA, Sigma-Aldrich, St. Louis, MI, USA) as the standard calibration curve in order to digest 30 µg of proteins for each treatment. On the other end, supernatants were tested for protein concentration through the Bradford assay and then, 30 µg of proteins was digested. In both cases, a tryptic digestion was carried out overnight at 37 °C. Protein label-free identification and quantification were performed in triplicate by LC-MS/MS with the UltiMate^TM^ 3000 UPLC (Thermo Fisher Scientific, Milan, Italy), using the already described method [19]. Briefly, tryptic peptides were analyzed through a Trap Cartridge C18 (0.3 mm ID, 5 mm L, 5 μm PS, Thermo Fisher Scientific) and then separated on an EASY-spray Acclaim^TM^ PepMap^TM^ C18 (75 μm ID, 25 cm L, 2 μm PS, Thermo Fisher Scientific) nanoscale chromatographic column. On the other hand, for supernatants and MIA-PaCa2 CM, the analytical method specifications were related to MS1 scans, which were performed in the Orbitrap analyzer through an HCD fragmentation as already described [20]. Raw proteomics data were deposited to the ProteomeXchange Consortium via the PRIDE partner repository [21] with the dataset identifier PXD030974.

### 2.5. Bioinformatics and Proteomics Data Processing

Proteomics MS/MS raw data were processed by using MaxQuant version 1.6.10.50 (Max-Planck Institute for Biochemistry, Martinsried, Germany). Andromeda was used for picking and peptide search was performed by the UniProt database (released 2018_04, taxonomy Homo Sapiens, 20,874 entries). As already reported in our previous works [19,20], carbamidomethylation of cysteines (C) was defined as fixed and quantification modification, while oxidation of methionines (M) and deamidation of asparagines (N) and glutamines (Q) were set as variable modifications. The 1% of both for protein and for peptide levels was chosen as the False discovery rate (FDR). LFQ Intensity was used to quantify protein abundance in each sample [22]. Bioinformatics analysis was performed as already reported [20]. Presence and absence of proteins as well as difference in protein expression were both evaluated for each comparison. Moreover, in order to define the differential proteins significantly expressed between the HDF cells or supernatants exposed to MIA-PaCa2 CM or DMEM SF at 24 h or 48 h, a univariate statistical analysis was performed with a *p*-value threshold of 0.05, visualizing results as a Volcano Plot. At least for each different comparison, protein ratios (HDF exposed to MIAPaCa-2 CM/HDF DMEM SF) were used for the Gene Ontology and functional enrichment analysis through the Ingenuity Pathway Analysis tool (IPA, Qiagen, Hilden, Germany), as already reported [20]. Briefly, IPA is able to predict the activation (z-scores ≥ 2.0) or inhibition (z-scores ≤ −2.0) of transcriptional regulators or downstreams for the loaded dataset based on prior knowledge of expected effects from published literature citations stored in the IPA system [23]. The STRING database (http://String-db.org/, accessed on 2 December 2021) was used to analyze PPI (protein-protein interaction) networks among the unique proteins found after different treatments. The confidence of predicted interactions was set at 0.4, indicating medium confidence.

### 2.6. Western Blot Analysis

Whole cell lysates were prepared using an ice-cold lysis buffer (TRIS-HCl 50 mM pH 8, NaCl 150 mM, Triton X-100 1%, NaF 100 mM, EDTA 1 mM, MgCl_2_ 1 mM, Glycerol 10%) supplemented with a protease inhibitor cocktail (Sigma-Aldrich, St. Louis, MO, USA) and a phosphatase inhibitor cocktail (Roche diagnostics, Mannheim, Germany). Protein content was assessed by the Bradford method (Bio-Rad). Equal amounts of proteins were separated on an SDS-PAGE and detected after being-transferred to a nitrocellulose membrane (GE life, Pittsburgh, PA, USA). Binding of non-specific antibodies was obtained by blocking the membrane with 5% not-fat dry milk in PBS with 0.01% Tween 20 for 1 h at RT and then incubated overnight with the following antibodies: anti-p44/42 MAPK (Erk1/2, MAPK3/1, 1:2000), anti-SMAD2/3 (1:1000), anti-GAPDH (1:1000) and anti-STAT3 (1:1000) (Cell Signaling Technology, Danvers, MA, USA); anti-LDHA (1:1000) and anti-alpha-SMA (1:500) (Thermo Scientific, Rockford, IL, USA); anti-ITGB3 used 1:1000 (Abcam, Cambridge, UK), anti-BAG3 used 1:2000 (Novus Biologicals, Milan, Italy) and anti-β-actin used 1:40000 (Sigma-Aldrich, Milan, Italy). Subsequently, the membrane was washed and incubated for 1 h at RT with the secondary antibody, diluted 1:20,000 (Bio-Rad, Milan, Italy). Bound antibodies were detected using the enhanced chemiluminescent (ECL) method (PerkinElmer Italia, Milan, Italy).

### 2.7. In Vitro Scratch Assay

A total of 250,000 HDFs were seeded on a six-well culture plate in fibroblast growth medium. After 24 h, the cell monolayer was scratched with a sterile 200 μL pipette tip. Then, HDFs were washed twice with PBS and fresh DMEM SF or MIA PaCa-2 CM was added. After performing the scratch (T0) and at 24 h and 48 h after treatment, images of the scratch area were taken with EVOS M5000 microscope (Thermo Fisher Scientific). The scratch area was measured using ImageJ software (software version 1.50i, nih.gov, Bethesda, MD, USA) and normalized to time T0 scratch. Two independent experiments were performed. Results are shown as mean ± standard deviation (SD). The multiple unpaired t test was chosen for analysis between two groups at different times using GraphPad Prism 9 (GraphPad Software, San Diego, CA, USA). The significance level was set at *p* < 0.05.

## 3. Results

### 3.1. Analysis of Protein Secreted by PDAC Cells

In order to investigate the mechanism by which the proteins secreted by pancreatic cancer cells contribute to the transformation of normal fibroblasts, we analyzed the MIA-PaCa2 conditioned medium (CM) using a quantitative label-free proteomics method.

We employed protocols without FBS supplement because it has been shown that low serum content conditions in combination with reduced biomechanical input lower the fibroblast activation state [24]. Moreover, the large number of proteins present in serum hampers in-depth proteomic analysis [25].

**Figure 1:**
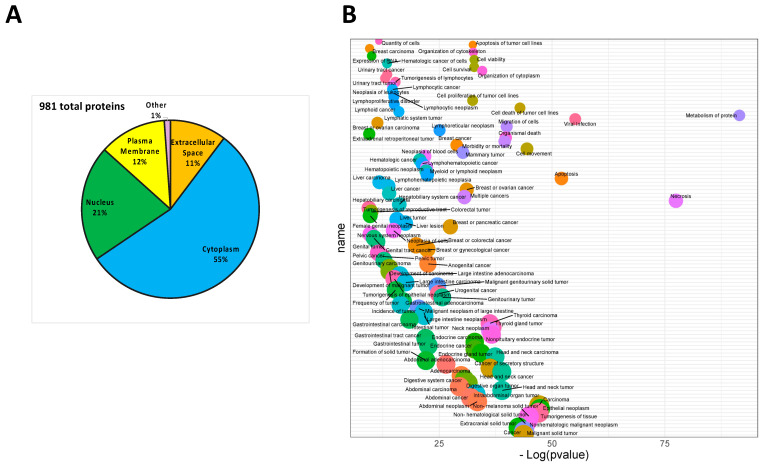
MIA-PaCa2 CM protein expression profile. (**A**) Subcellular localization of proteins identified in MIA-PaCa2 CM represented by pie chart. (**B**) Biological processes associated with proteins identified in MIA-PaCa2 CM represented by bubble plot. Ingenuity pathway analysis was used to categorize the proteins based on their functional assignments.

A total of 981 proteins were identified in MIA-PaCa2 CM (Figure 1A), of which 55% were cytoplasmic proteins, 21% were nuclear, 12% were known to be membrane bound and 11% were extracellular proteins (Figure 1A). This seemingly improper protein distribution is due not to cellular residues in the CM, but most likely to the presence of microvesicles in these supernatants as reported in our previous study, as well as to proteins released following cell death [26,27]. According to IPA functional analysis, the proteins identified are involved in protein metabolism (*p*-value = 2.97 × 10^−92^), necrosis (*p*-value = 3.56 × 10^−78^), viral infection (*p*-value = 7.30 × 10^−56^) and apoptosis (*p*-value = 8.22 × 10^−53^) (Figure 1B). The complete list of molecular functions related to the identified proteins is reported in Supplementary Table S1.

### 3.2. Effect of MIA-PaCa2 Conditioned Media on HDFs

CM of MIA-PaCa2 were used to treat HDFs for 24 h and 48 h as detailed in the methods section and both cell lysates and supernatants were analyzed by proteomics.

**Figure 2:**
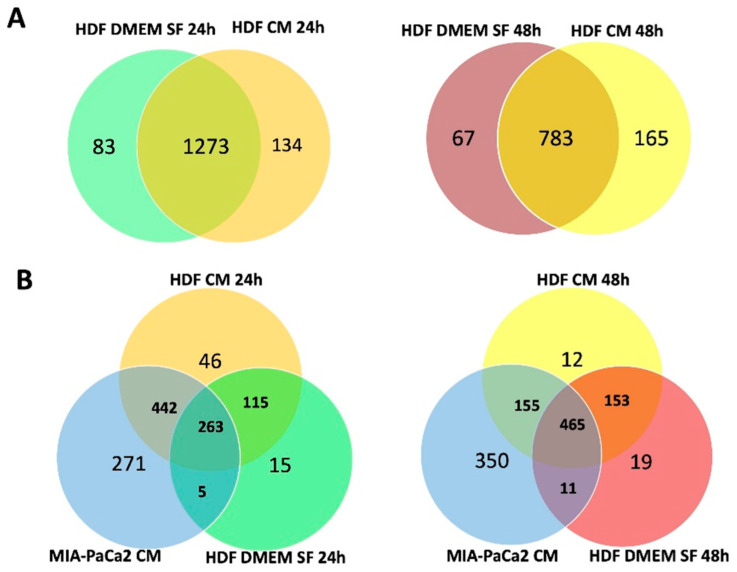
Proteins identified in HDF cell lysates and supernatants. (**A**) Venn diagrams showing the number of proteins detected in HDF total cell lysates treated (HDF CM) or not (HDF DMEM SF) with MIA-PaCa2 CM for 24 h and 48 h. Proteins in common between treated and untreated samples are indicated by the overlapping circles. (**B**) Venn diagrams showing the number of proteins detected in HDF supernatants treated (HDF CM) or not (HDF DMEM SF) with MIA-PaCa2 CM for 24 h and 48 h. Proteins in common between samples are indicated by the overlapping circles.

In HDF cell lysates treated for 24 h with MIA-PaCa2 CM, we identified a total of 1490 proteins of which 73 were characteristic of the control (untreated fibroblasts), 136 were characteristic of the treated sample and 1273 were in common (Figure 2A). In the 48 h treated samples, we identified a total of 1015 proteins of which 67 were characteristic of the control, 165 were characteristic of the treated sample and 783 were in common (Figure 2A).

In HDF supernatants treated with MIA-PaCa2 CM, we identified a total of 1157 proteins after 24 h of treatment and 1165 proteins after 48 h. Some of these proteins were only present in the HDF supernatants after treatment (46 after 24 h and 12 after 48 h) and some were no longer detectable following treatment (15 after 24 h and 19 after 48 h) (Figure 2B).

A quantitative analysis of differentially expressed (DE) proteins revealed 125 significantly downregulated and 78 significantly upregulated proteins in HDF cell lysate treated for 24 h with MIA-PaCa2 CM (Supplementary Figure S1A), whereas after 48 h of treatment, 107 proteins were downregulated and 117 were upregulated (Supplementary Figure S1A).

In the HDF supernatants treated with MIA-PaCa2 CM, 94 proteins were downregulated and 143 were upregulated after 24 h of treatment, whereas after 48 h of treatment, 119 proteins were downregulated and 247 were upregulated (Supplementary Figure S1A).

To validate the proteomics results, we analyzed the expression of mitogen activated protein kinase 1/3 (MAPK1/3) and lactate dehydrogenase A (LDHA) by Western blotting, known to be activated in CAFs as reported by several studies [28,29,30]. Both proteins, MAPK1/3 and LDHA, were markedly downregulated after 24 h of treatment and upregulated after 48 h of treatment (Supplementary Figure S1A). Western blot analysis confirmed the reduced expression of MAPK1/3 and LDHA in HDF cells treated for 24 h and their overexpression after 48 h of treatment (Supplemetary Figure S1B).

Proteomics data were analyzed by IPA software to generate an overview of the major biological pathways switched on and off in HDFs treated with MIA-PaCa2 CM.

**Figure 3:**
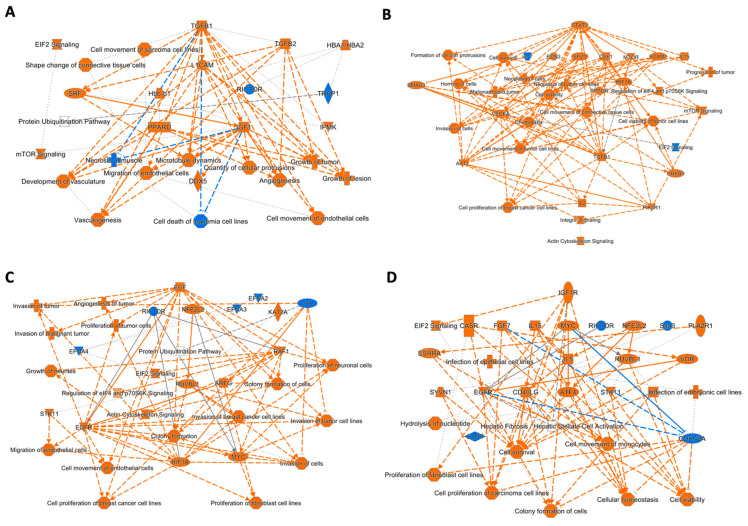
Graphical summaries of IPA pathway analysis. Nodes significantly upregulated (orange) and downregulated (blue) in HDF cell lysates after 24 h (**A**) and 48 h (**B**) of treatment with CM. Nodes significantly upregulated (orange) and downregulated (blue) in HDF supernatants after 24 h (**C**) and 48 h (**D**) of treatment with CM.

In Figure 3, IPA graphical summaries show hierarchical representations of the possible molecular mechanisms and key functional effects induced by each treatment on cell lysates and HDF supernatants.

**Figure 4:**
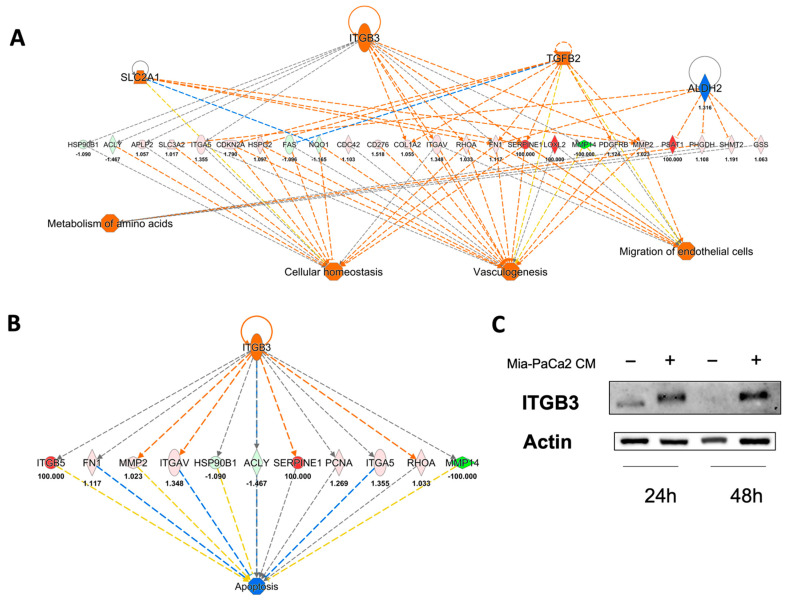
Upstream regulators engaged by 24 h of treatment with MIA-PaCa2 CM. (**A**) Network analysis showing the upstream regulators predicted to be activated (SLC2A1, ITGB3 and TGFB2) and inhibited (ALDH2). The downstream biological effects are also shown. (**B**) Activated ITGB3 is predicted to inhibit apoptosis. Orange node and edges indicate activation, while blue nodes and edges indicate inhibition. Yellow edges reveal an inconsistent relationship between the findings and the state of the downstream node; instead, the gray ones indicate no predicted effects. (**C**) Western blot confirming upregulation of ITGB3.

The most evident effect after 24 h of treatment with the CM on HDF cells was the activation of pathways leading to cell growth, motility and angiogenesis (Figure 3A). Such effects involve the activation of TGFB1 and 2, L1 cell adhesion molecule (L1CAM), serum response factor (SRF), peroxisome proliferator-activated receptor delta (PPARD) and insulin-like growth factor I (IGF1) as the most representative molecules (Figure 4A).

After 48 h of treatment, in addition to cell proliferation and invasion, we observed also the activation of actin cytoskeleton signaling (Figure 4B). These functions involve the activation of STAT3, macrophage colony-stimulating factor 1 (CSF1), serine/threonine-protein kinase mTOR (MTOR), Forkhead box protein M1 (FOXM1) and vascular endothelial growth factor A (VEGFA) as the most representative molecules (Figure 3B).

Analysis of DE proteins released from HDFs in the supernatant after treatment with MIA-PaCa2 CM for 24 h confirmed a possible effect on cell proliferation, migration, and invasion (Figure 3C). The proteins involved in the control of such functions might be the epidermal growth factor (EGF), nuclear factor erythroid 2-related factor 2 (NFE2L2), RAF proto-oncogene serine/threonine-protein kinase (RAF1), RuvB-like 1 (RUVBL1), amphiregulin (AREG), Myc proto-oncogene protein (MYC), epidermal growth factor receptor (EGFR) and hypoxia-inducible factor 1-alpha (HIF1A) (Figure 3C). Finally, after 48 h of treatment, HDF supernatants still displayed a repertoire of DE proteins involved in increasing cell survival, cell viability and regulatory mechanisms to preserve cellular homeostasis (Figure 3D). Major players involved in these processes possibly include IGF 1, MYC and NFE2L2 (Figure 3D).

### 3.3. Regulator Effects Predicted by IPA in HDF Treated Cell Lysates

Regulator effect analysis enables identification of which protein is activated or inhibited upstream and what kind of biological activities are affected downstream in a given experimental dataset. The complete lists of predicted upstreams and downstreams are displayed in Supplementary Table S2.

**Figure 5:**
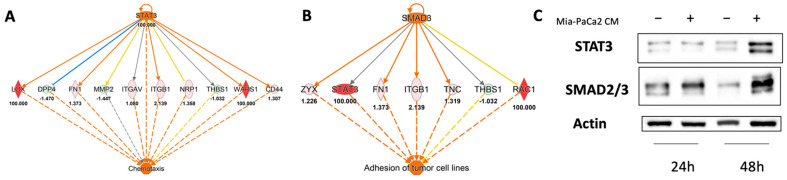
Upstream regulators engaged by 48 h of treatment with MIA-PaCa2 CM. Predicted upstream regulators were STAT3 (**A**) and SMAD3 (**B**). (**C**) Western blot confirming the involvement of STAT3 and SMAD3 as predicted by IPA analysis.

This analysis, performed on an HDF cell lysate treated for 24 h with the CM of MIA-PaCa-2, confirmed the activation of cell migration and angiogenesis as well as revealed an improvement of cell homeostasis and metabolism of amino acids (Figure 5A). ITGB3, Solute carrier family 2 member 1 (SLC2A1) and TGFB2 are predicted to be the activated regulators; instead, mitochondrial aldehyde dehydrogenase, (ALDH2) is predicted to be an inhibited regulator (Figure 5A). To control cell migration and angiogenesis, ITGB3 acts on different targets including alpha-1 (I) chain of collagen (COL1A1), integrin alpha-5 (ITGA5), transforming protein RhoA (RHOA), plasminogen activator inhibitor 1 (SERPINE1) and matrix metallopro-teinase-2 (MMP2) (Figure 5A). TGFB2 acts upstream of eight proteins, including cyclin dependent kinase inhibitor 2A (CDKN2A), integrin subunit alpha V (ITGA5), RHOA, platelet derived growth factor receptor beta (PDGFRB), MMP2, and SERPINE1 (Figure 5A). Note that both regulators (ITGB3 and TGFB2) converge on a few common intermediates (Figure 4A). Finally, ITGB3 was also predicted to be involved in apoptosis inhibition via MMP2, ITGAV, ATP-citrate lyase (ACLY), SERPINE1 and RHOA (Figure 4B). Western blot analysis confirmed the predicted overexpression of ITGB3 after 24 h of treatment and showed a clear upregulation of ITGB3 also after 48 h of treatment (Figure 4C).

Next, we investigated the upstream regulators in HDF cell lysates activated after 48 h of treatment with MIA-PaCa2 CM. STAT3 and SMAD3 were significantly activated (Figure 5A,B) and Western blot analysis confirmed this IPA prediction (Figure 5C). STAT3 possibly controls chemotaxis though a functional relationship with integrins (ITGAV, integrin beta 1 (ITGB1)) and ECM (lysyl oxidase (LOX), fibronectin 1 (FN1), MMP2, thrombospondin 1 (THBS1)) (Figure 5A). SMAD3 might regulate cell adhesion by acting on a few targets in common with STAT3, including STAT3 itself (Figure 5B).

Finally, using the STRING database, we studied PPIs occurring among proteins identified exclusively in cell lysates or supernatants of CM-treated HDF cells but not in untreated control fibroblasts.

**Figure 6:**
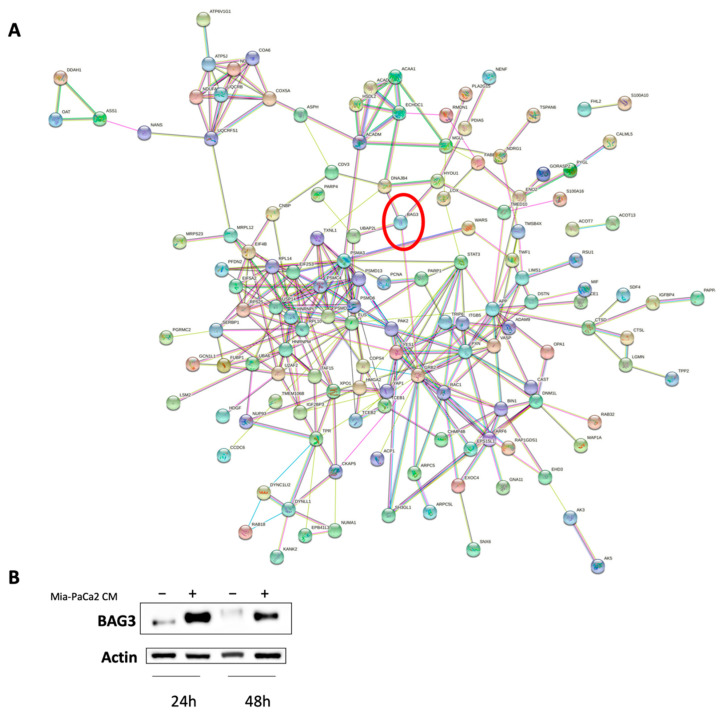
BAG3 is strongly induced in HDF cells treated with MIA-PaCa-2 CM. (**A**) PPI network predicted by STRING among the proteins identified in lysate of HDF cells treated for 48 h with CM. BAG3 is highlighted by a red circle. (**B**) Western blot of BAG3 in HDF cell lysate treated for 24 h and 48 h with MIA-PaCa-2 CM.

As described above, 134 and 165 distinct proteins were identified in HDF cell lysate after 24 h and 48 h of treatment (Figure 2A). Instead, in HDF supernatants there were only 46 and 12 proteins (Figure 2B). Among the proteins exclusively identified in the cell lysate of HDF treated for 48 h was BAG3. Notably, BAG3 was expressed along with four of its interactors, namely hypoxia up-regulated 1 (HYOU1), growth factor receptor-bound protein 2 (GRB2), ubiquitin-associated protein 2-like (UBAP2L) and DNAJ heat shock protein family (Hsp40) member B4 (DNAJB4) (Figure 6A). We confirmed BAG3 expression by Western blot analysis and found that it was also upregulated after 24 h of treatment (Figure 6B).

### 3.4. MIA-PaCa2 CM Increases HDFs Migration

**Figure 7:**
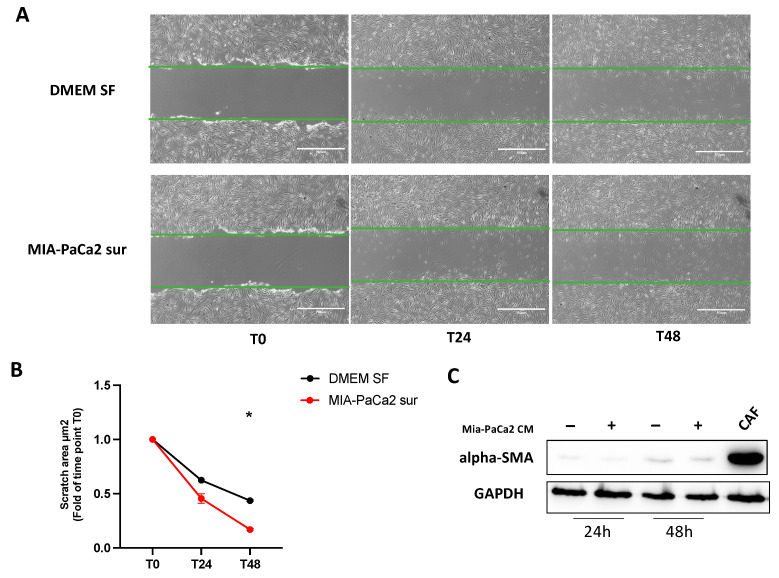
Increased migration in HDF cells treated with MIA-PaCa-2 CM. (**A**) Representative images of scratch assay at different time points (T0, T24 and T48). The scratch is highlighted in green. Scale bar, 750 µm. The wound closure area was calculated with ImageJ software. (**B**) Relative scratch area closure normalized to time T0 scratch. Data represent the means ± SD of two independent experiments. Symbol indicates significant difference between DMEM SF and treated cells (* *p* < 0.05). (**C**) Western blot analysis of alpha-SMA expression after 24 and 48 h of treatment compared to CAFs as positive control.

A scratch assay was performed in order to validate proteomics results regarding enhanced migration in HDFs after treatment with MIA-PaCa2 CM. As shown in Figure 7A,B, treated HDFs resulted in faster wound closure compared to the control at 24 h and 48 h after treatment, in accordance with the increased cell movement function found by IPA. In addition, we analyzed alpha-SMA expression to monitor HDF activation state and we noticed that HDFs treated with CM exhibited a low contractile phenotype when compared to CAFs used as an alpha-SMA positive control (Figure 7C).

## 4. Discussion

Despite extensive research having been conducted in delineating the role of fibroblasts in pancreatic ductal adenocarcinoma progression, the role of these cells in earlier tumor development is still incompletely understood. To date, cellular crosstalk between pancreatic cancer cells and stromal cells appears to be a critical point. Abnormal proliferation of fibroblasts and increased deposition of ECM create an environment that facilitates tumor growth, metastasis and drug resistance. Therefore, it is important to discover the events occurring in the tumor microenvironment, especially in the initial activation of normal fibroblasts.

Although it is difficult, in vitro, to reproduce the complexity of the tumor microenvironment, in the present study, using LC-MS/MS, we investigated the initial changes that occur in normal fibroblasts treated with the conditioned medium of pancreatic cancer cells for 24 h and 48 h. To avoid possible secondary sources of HDF activation, we used protocols without FBS supplement. Our proteomic analysis of normal fibroblasts treated with MIA-PaCa2 CM identified cell movement as one of the key biological functions activated after both 24 h and 48 h of treatment confirmed by an in vitro scratch assay. Moreover, the low expression of alpha-SMA in treated HDFs compared to CAFs highlighted a more migratory capacity instead of an increase in cell contractility found to be in accordance with previous studies [31,32,33]. However, Wipff et al. reported that the conjunctive presence of mechanical stress and active TGFBeta is essential to convert fibroblasts into contractile myofibroblasts [32]. Even if in our experiments, TGFbeta was predicted by IPA analysis to increase both after 24 h and 48 h of treatment, the expression of alpha-SMA was still not observed, possibly due to the absence of mechanical stress.

Furthermore, we observed an increase in cellular homeostasis and vasculogenesis, while apoptosis was inhibited, in line with a number of previous reports. ITGB3 and TGFBeta2 are the predicted upstream activators involved in the response of HDF cells treated for 24 h, whereas SMAD3 and STAT3 are the predicted upstream activators after 48 h of treatment. Integrins, acting like a bridge between the extracellular matrix and the intracellular cytoskeleton, can transduce a variety of signals from the extracellular matrix and affect cell survival and differentiation [34]. While SMAD3 and STAT3 transmit signals from plasma membrane receptors to the nucleus, STAT3 is a key signal transducer of the cytokine receptors, including IL-6, leukemia inhibitory factor (LIF), and oncostatin M. SMAD3 operates downstream of growth/differentiation factors such as TGF beta, one of the key molecules predicted to increase both after 24 h and 48 h of treatment. Accumulating evidence indicates that SMAD3 and STAT3, in a highly context-dependent manner, can interact and cooperate [35]. STAT3 functions as a positive regulator in TGFBeta1-induced epithelium-mesenchymal transition (EMT) and metastases in hepatocellular carcinoma (HCC). STAT3 and Snail-Smad3/TGFBeta1 signaling pathways synergistically increase EMT and migration of HCC [36].

We also suggest that the initial activation of fibroblasts may also be caused by a metabolic reprogramming, in line with O’Leary et al. demonstrating that TGFBeta induces metabolic reprogramming in lung fibroblasts characterized by increased levels of glycolysis and mitochondrial oxygen consumption [37].

Finally, we discovered the presence of BAG3 among the proteins induced in HDFs treated for 48 h with CM. BAG3 expression was confirmed by Western blotting in cells treated for 24 h and in those treated for 48 h. BAG3 has been associated to a variety of pathophysiological processes including autophagy, aggresome formation, cell transformation and survival [38]. The aberrant expression of BAG3 has been reported to contribute significantly to the reprogramming of glucose metabolism in PDAC cells [39]. Furthermore, BAG3 overexpression has been associated with actin reorganization and increased cell motility of several human tumor cell lines [40].

Our STRING analysis revealed that four BAG3 interactors are induced in normal fibroblast cells after CM treatment. This hypothetical functional complex consisting of BAG3, HYOU1, GRB2, UBAP2L and DNAJB4 could be very important in PDAC since, in addition to BAG3, interactors also play an important role in tumor pathology. Specifically, HYOU1 is an endoplasmic reticulum chaperone protein known to be induced by a variety of stress conditions including hypoxia, glucose deficiency, reducing agents and tunicamycin. HYOU1 inhibits apoptosis, maintains calcium homeostasis, and exhibits cytoprotective effects by preventing endoplasmic reticulum stress [41]. GRB2 is a key molecule in intracellular signal transduction, linking activated cell surface receptors to downstream targets by binding to specific phosphotyrosine-containing and proline-rich sequence motifs. We suggest that BAG3 interacting with GRB2 could improve actin-based cell motility, as well as more complex processes such as epithelial morphogenesis, angiogenesis and vasculogenesis, as reported by Giubellino et al. [42]. UBAP2L, a highly conserved protein that contains an N-terminal ubiquitin-associated (UBA) domain, is part of the ubiquitin-proteasome system. Recent studies have confirmed that UBAP2L functions as an oncogene and is associated with various types of cancer [43,44]. Finally, DNAJB4 is a DNAJ family protein for which tumor suppressor activity has been demonstrated in breast, lung and gastric cancers [45,46,47].

In conclusion, our findings provide new insight into the mechanisms that contribute to the earliest activation of normal fibroblasts in the PDAC microenvironment. In particular, we identified a repertoire of proteins induced in fibroblasts treated with the conditioned medium of MIA-PaCA2. Data mining of their proteomics indicated that ITGB3, TGFB1 and TGFB2 could be key players in increasing fibroblast movement and inhibiting apoptosis, whereas SMAD3, STAT3 and BAG3 could be important molecules in activating chemotaxis, cell adhesion and actin cytoskeleton signaling.

## Data Availability

The data presented in this study are available in this article.

## Supplementary Materials

The following supporting information can be downloaded at: https://www.mdpi.com/article/10.3390/cells11071160/s1. Supplementary Tables S1 and S2 and Supplementary Figure S1. Graphical abstract was created with BioRender.com Software (2022).
