# Supplementary material for: Proteomics Approach Highlights Early Changes in Human Fibroblasts-Pancreatic Ductal Adenocarcinoma Cells Crosstalk
Source: Cells. 2022 Mar 29;11(7):1160. doi: 10.3390/cells11071160 (PMC8997741; doi:10.3390/cells11071160)
Supplement: Supplementary file 1 [file cells-11-01160-s001.zip › Supplementary Figure S1.pdf]

Supplementary Materials of:

Proteomics approach unravels early changes in Human Fibroblasts-Pancreatic Ductal Adenocarcinoma cells crosstalk

Verena Damiani [1,2], Maria C. Cufaro [2,3], Maurine Fucito [1,2], Beatrice Dufrusine [1,2], Claudia Rossi [1,4], Piero Del Boccio [2,3], Luca Federici [1,2], Maria C. Turco [5,6], Michele Sallese [1,2], Damiana Pieragostino [1,2,*] and Vincenzo De Laurenzi [1,2]

[1] Department of Innovative Technologies in Medicine and Dentistry,University "G. d'Annunzio" of Chieti- Pescara, Italy; verena.damiani@unich.it (V.D.); maurine.fucito@unich.it (M.F.); beatrice.dufrusine@unich.it (B.D.); luca.federici@unich,it (L.F.); michele.sallese@unich.it (M.S.); damiana.pieragostino@unich.it (D.P.); delaurenzi@unich.it (V.D.L.)

[2] Center for Advanced Studies and Technology (CAST), University "G. d'Annunzio" of Chieti-Pescara, Italy

[3] Department of Pharmacy, University "G. d'Annunzio" of Chieti-Pescara, Chieti, Italy; maria.cufaro@unich.it (M.C.C.); piero.delboccio@unich.it (P.D.B.)

[4] Department of Psychological, Health and Territory Sciences, School of Medicine and Health Sciences, "G. d'Annunzio" University of Chieti-Pescara, 66100 Chieti, Italy; claudia.rossi@unich.it (C.R.)

[5] Department of Medicine, Surgery and Dentistry Schola Medica Salernitana, University of Salerno, Baronissi, Italy; mcturco@unisa.it (M.C.T.)

[6] R&D Division, BIOUNIVERSA s.r.l., Baronissi, Italy

* Correspondence: damiana.pieragostino@unich.it; Tel: +39 0871541593

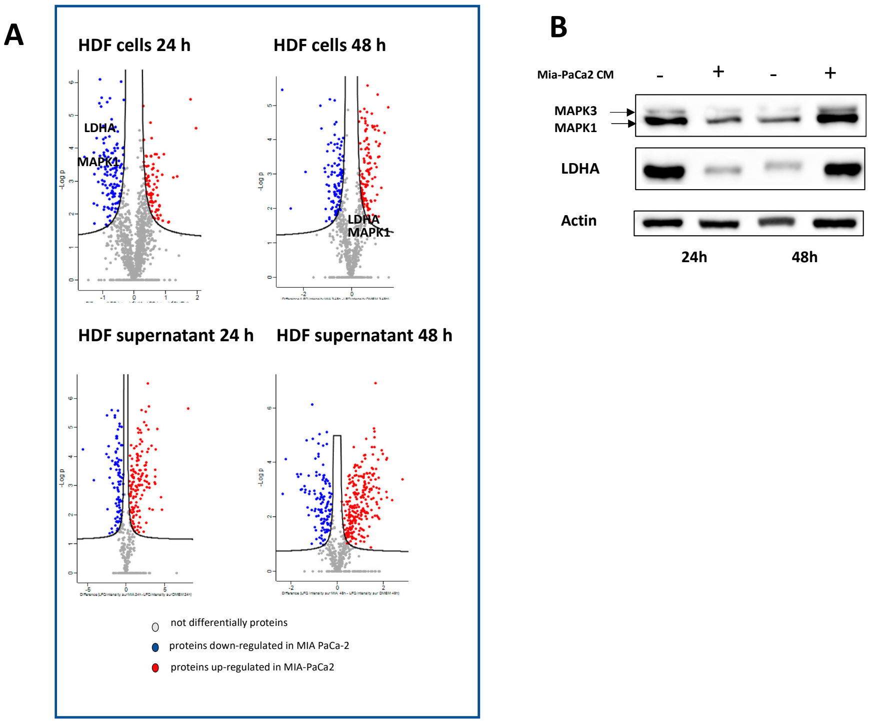

Supplementary Figure S1. Validation of MS/MS data. (A) Volcano plots of DE proteins identified by MS/MS analysis in lysate and supernatant of HDF cells treated for 24 and 48 hours with MIA-PaCa2 CM. Downregulated (24 hours) and upregulated (48 hours) MAPK1/3 and LDHA are indicated. (B) Western blot analysis of MAPK1/3 and LDHA in cell lysates of HDF cells treated with MIA-PaCa2 CM.
